# Acute Renal Infarction After a Bilateral Aortic-Iliac Stent Thrombosis

**DOI:** 10.7759/cureus.53211

**Published:** 2024-01-30

**Authors:** Beatriz Chambino, Cláudio Gouveia, Cristiana Camacho, Antony Dionisio, Ana Margarida Ribeiro, Célia Henriques

**Affiliations:** 1 Internal Medicine, Hospital São Francisco Xavier, Lisbon, PRT; 2 Internal Medicine, Centro Hospitalar Lisboa Ocidental, Lisbon, PRT; 3 Internal Medicine, Heart Failure Clinic, Lisbon, PRT

**Keywords:** aortic-iliac stent, renal artery infarction, high blood pressure, abdominal ct angiography, sustained low-efficiency dialysis, nephritic syndrome

## Abstract

A renal infarction occurs when kidney's arterial blood supply is compromised, causing parenchymal necrosis and loss of function. It is a relatively uncommon complication and its treatment is time-dependent. We present a case where a female patient with a history of bilateral aortic-iliac stenting over 10 years before presented with chest pain, palpitations, and dyspnea associated with hypertension. The patient progressed with an acute worsening of renal function and anuria, with an urgent need for renal replacement therapy. The abdominal CT angiography confirmed a complete chronic stent thrombosis and a recent occlusion of the right renal artery causing an acute renal infarction; however, this exam was performed more than 72 hours after admission. There was no longer indication for reperfusion therapy, taking into account the time course. This case reinforces the importance of a thorough clinical history and awareness of risk factors to raise the suspicion of renal infarction that should lead to an early contrast-enhanced CT scan so that adequate therapy can be performed.

## Introduction

Acute renal infarction consists of an ischemic event caused by the occlusion (complete or partial) of the main renal artery or its branches, leading to renal parenchyma damage and eventual necrosis [1,2]. It is a presumed uncommon cause of acute kidney failure; however, the true incidence is unknown due to a lack of awareness and difficulty in diagnosing [1].

Clinical manifestations are non-specific and could delay the correct diagnosis [3,4]. The sudden onset of diffuse abdominal or flank pain, fever, nausea, and hematuria are some of the typical symptoms. Laboratory findings may include leukocytosis, elevated C-reactive protein (CRP), and lactate dehydrogenase (LDH) [2,3]. Elevation of creatinine level and a decline in glomerular filtration rate (GFR) are only visible with a particularly large renal infarct or involvement of both kidneys [1,3].

There are two major causes: embolism from the heart or aorta and in-situ thrombosis [1,4]. Other causes are coagulation disorders, vasculitis, fibromuscular dysplasia, aortic aneurysms, and trauma [4]. A renal CT arteriography is the gold standard of investigation for renal infarction [1,2].

Once the diagnosis is established, prompt revascularization therapy with fibrinolytics or anticoagulants may reverse ischemia and preserve renal function [3]. The effectiveness of reperfusion therapy is time-dependent, and a delayed diagnosis could lead to irreversible loss of renal function [1]. In a complete main renal arterial occlusion, revascularization should be performed under a six-hour time window, and in partial arterial occlusion, therapy could be beneficial for up to 24 hours [1].

We present a case of progressive renal infarction caused by chronic thrombosis of bilateral aortic-iliac stenting in place for more than 10 years. A complex clinical picture, complicated by type 3 cardio-renal syndrome [5], caused a delay in diagnosis. At the time of the definitive diagnosis, there was no longer indication for reperfusion therapy, and the patient needed transitory renal function replacement therapy [2]. A thorough clinical history and a high index of suspicion are essential for diagnosing an acute renal infarction [4].

## Case presentation

A 65-year-old Caucasian female presented to the emergency department following an episode of palpitations, dyspnea and chest pain of acute onset, and short duration and spontaneous resolution.

Her medical history included active heavy smoking (one pack per day for 48 years), peripheral arterial disease (with bilateral aortic-iliac stenting, in Switzerland, back in 2012) managed with long-term clopidogrel and a recent episode of deep venous thrombosis having been on rivaroxaban for a six-month period during the previous year. At the time of admission, the patient was asymptomatic and her blood pressure was 228/138 mmHg. Physical examination was unremarkable except for fine crackles within the lower pulmonary lobes and bilateral symmetric edema of the legs. Electrocardiogram at admission revealed discreet alterations of ventricular repolarization compatible with systolic overload. Arterial blood gas results showed hypoxemia and respiratory alkalosis. Chest radiograph demonstrated bilateral pleural effusion. Laboratory tests revealed elevated NTproBNP (3625 pg/mL), LDH (309 U/ L), and D-dimer (2332 μg/mL) levels; normal urea, electrolytes and creatinine and no variation of troponin concentration on serial evaluation. Pulmonary CT angiography showed no evidence of pulmonary thromboembolism.

A diagnosis of new-onset heart failure with resulting bilateral pleural effusion and partial respiratory insufficiency was made, and the patient was treated with intravenous (IV) diuretics and supplemental oxygen therapy. Transthoracic echocardiography revealed a slightly reduced left ventricular function and a normal inferior vena cava diameter with an inspiratory collapse >50%; therefore, diuretic therapy was progressively reduced.

During the first hours of hospitalization, the patient developed nausea, vomiting, and acute right flank abdominal pain. Urgent abdominopelvic ultrasound and CT scan were unremarkable. The patient maintained high blood pressure with progressive deterioration of consciousness despite a continuous increase in antihypertensive medication. A cranial CT scan showed no evidence of cerebral edema or any other acute intracranial pathology.

Additionally, continuous worsening of renal function with decreased urinary output was noted despite fluid administration. Urinalysis showed microscopic hematuria and proteinuria, consistent with nephritic syndrome. Renal ultrasound results suggested acute medical nephropathy. Hepatitis B, C, and human immunodeficiency virus serologies were negative. Workup for autoimmune and neoplastic disorders was negative. The patient was started on sustained low-efficiency dialysis (SLED) for acute kidney injury with progressive oligoanuria and maintained treatment for six days.

**Figure 1:**
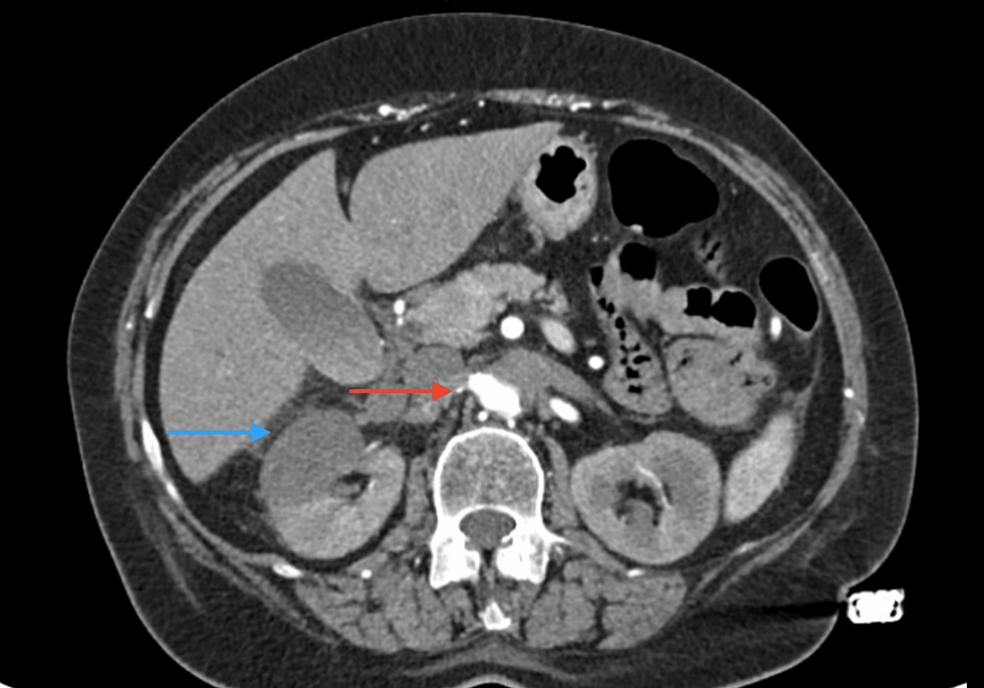
Abdominal CT angiography confirming an extensive opacification defect of the right renal artery (red arrow) and a parenchymal infarction (blue arrow), confirming the diagnosis of a recent renal infarction.

**Figure 2:**
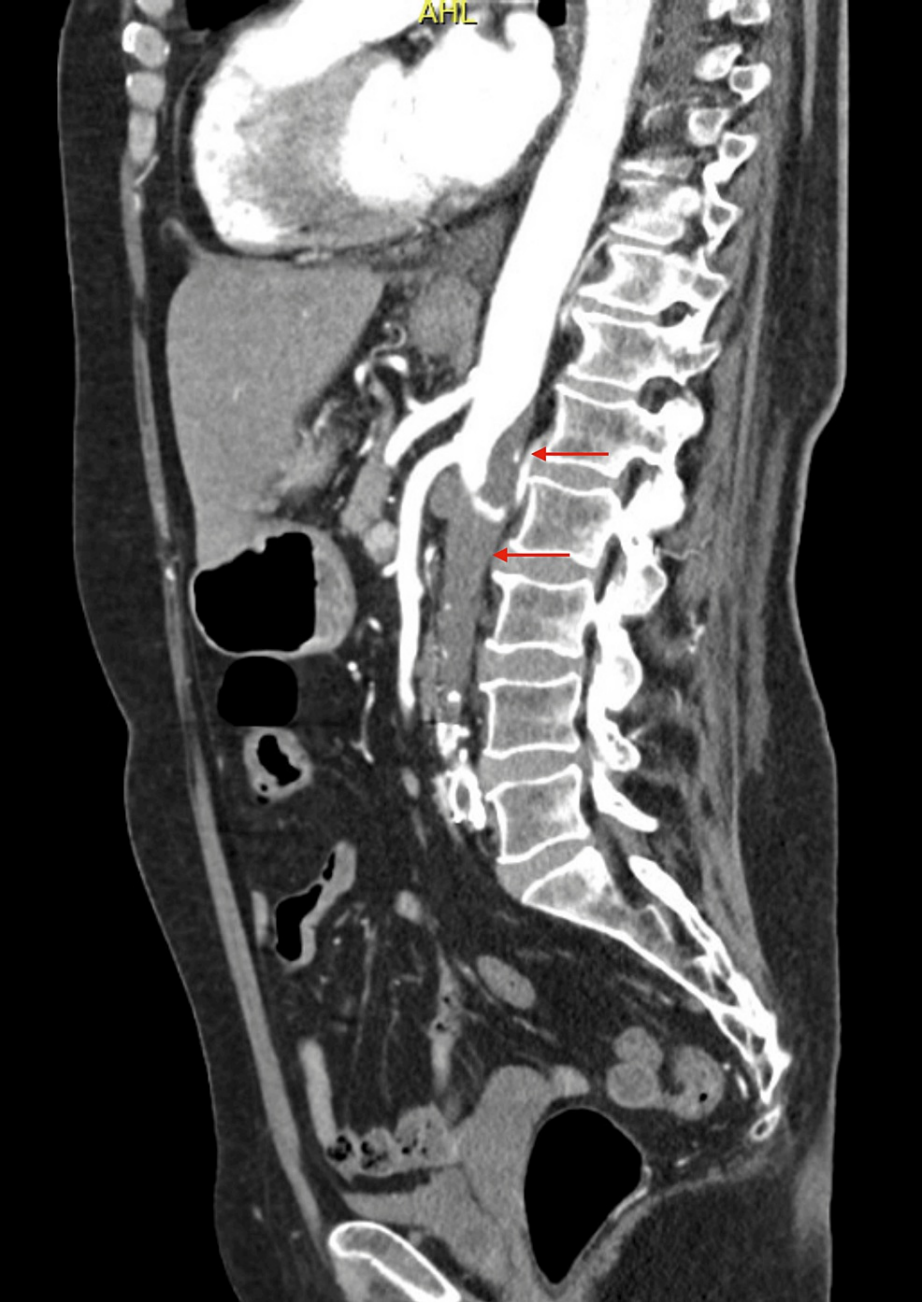
Abdominal CT angiography showing an almost complete opacification defect of the abdominal aorta (red arrows), below renal branches, with features suggesting chronic stent thrombosis.

On the second day, given the patient’s past medical history of bilateral aortic-iliac stenting over 10 years before, the examining nephrologist suspected acute renal infarction, and an abdominal CT angiography (Figures 1, 2) confirmed an almost complete opacification defect of the abdominal aorta and left renal artery, with features suggesting chronic stent thrombosis, as well as an extensive opacification defect of the right renal artery, confirming the diagnosis of recent renal infarction concerning the anterior and superior portion of the right kidney. This progressive renal infarction could justify the worsening of blood pressure control and the clinical manifestations of acute heart failure, assuming a possible type 3 cardio-renal syndrome.

**Figure 3:**
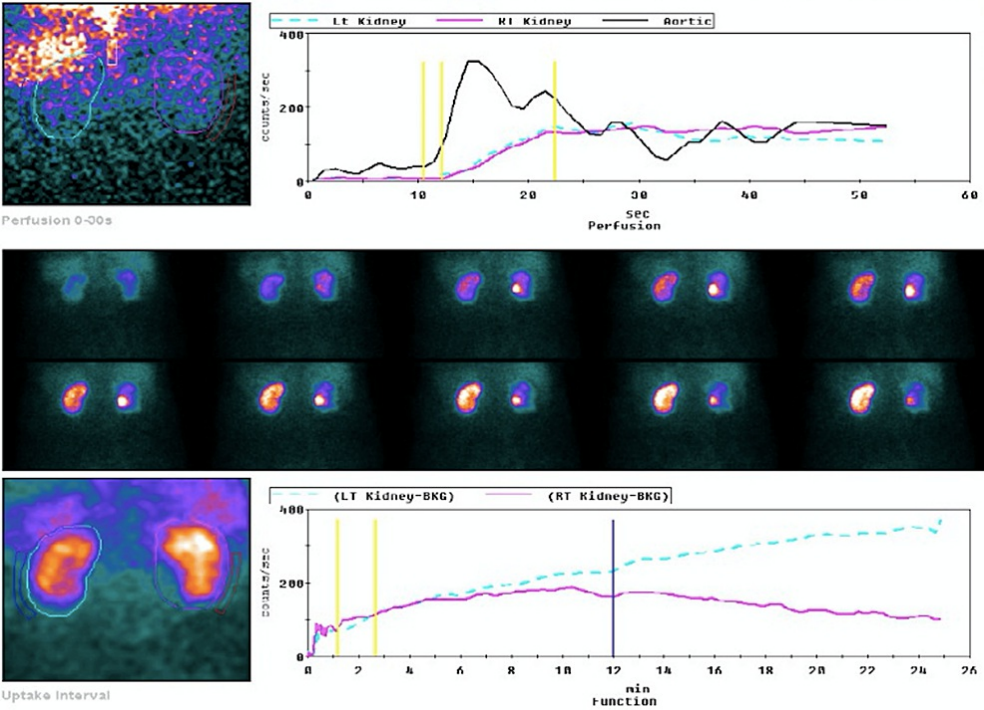
MAG3 renogram showing a symmetric but severely reduced tubular renal function. Significant parenchymal retention of the radiopharmaceutical in the left kidney is suggestive of obstruction to drainage.

The case was discussed with vascular surgery; however, given the time course, there was no indication for invasive procedures. Following diagnosis, the patient was treated with low-molecular-weight heparin to maintain anti-Xa activity within a target therapeutic range. A follow-up MAG3 renogram (Figure 3) showed symmetric but severely reduced tubular renal function. After SLED was suspended, renal scintigraphy results revealed a scarred left kidney and an almost normal radioisotope uptake in the right kidney. Urine output was restored, and the patient continued in-hospital hemodialysis for two more weeks. In this case, anticoagulant and antiplatelet therapy were introduced.

## Discussion

Renal infarction is a rare diagnosis [1,4] and is frequently misinterpreted as pyelonephritis, nephrolithiasis, or other extra-renal disorders [1-4]. Risk factors, such as age over 70 years, atrial fibrillation, serum LDH >500 IU/L, diabetes and hypertension, might raise suspicion of renal infarction [1,3]. At first glance, this patient could have new-onset heart failure with uncontrolled blood pressure, but the rapid deterioration of renal function could not be explained.

The patient’s clinical history, in this specific case, was difficult to unravel because this vascular procedure happened in a foreign country and quite a long time before this episode. Although endovascular treatment for peripheral artery disease is proven to be safe and effective in the long term, it still has a risk of restenosis and loss of patency [6]. Hypercoagulable states are known to cause renal infarction in less than 10% of cases [4]. However, there is no case reporting acute renal infarction with this type of endovascular therapy.

This patient was subjected to several imaging exams (two ultrasound scans and one non-contrast CT scan) that excluded most of the frequent causes for the clinical manifestations present. However, an abdominal CT angiography was only performed 72 hours after renal function declined. A CT angiography is the definitive exam to confirm the diagnosis and should be performed within six hours from presentation in complete artery occlusions and within 24 hours in partial artery occlusions to ensure the effectiveness of revascularization [1]. However, more than two days of delays in achieving the correct diagnosis are common in 50% of cases, directly influencing the prognosis [1]. When arterial reperfusion is not indicated, anticoagulation therapy as well as strict control of cardiovascular risk factors are essential [4].

## Conclusions

This case report allowed us to raise awareness about the most basic and simple action when attending a patient: an exhaustive past clinical history. We can approach differential diagnoses only with all the information and risk factors. We reinforce the indication for a contrast-enhanced CT scan within 24 hours in all patients with high risk for acute renal infarction.
